# Genome assembly of the acoel flatworm *Symsagittifera roscoffensis*, a model for research on body plan evolution and photosymbiosis

**DOI:** 10.1093/g3journal/jkac336

**Published:** 2022-12-21

**Authors:** Pedro Martinez, Kirill Ustyantsev, Mikhail Biryukov, Stijn Mouton, Liza Glasenburg, Simon G Sprecher, Xavier Bailly, Eugene Berezikov

**Affiliations:** Departament de Genètica, Microbiologia i Estadística, Universitat de Barcelona, Av. Diagonal 643, 08028 Barcelona, Spain; Institut Català de Recerca i Estudis Avançats (ICREA), Barcelona 08193, Spain; European Research Institute for the Biology of Ageing, University Medical Center Groningen, University of Groningen, Groningen 9700AD, The Netherlands; Institute of Cytology and Genetics SB RAS, Novosibirsk 630090, Russia; European Research Institute for the Biology of Ageing, University Medical Center Groningen, University of Groningen, Groningen 9700AD, The Netherlands; European Research Institute for the Biology of Ageing, University Medical Center Groningen, University of Groningen, Groningen 9700AD, The Netherlands; Department of Biology, University of Fribourg, Chemin du Musee 10, 1700 Fribourg, Switzerland; Station Biologique de Roscoff, Multicellular Marine Models (M3) team, FR2424, CNRS/Sorbonne Université—Place Georges Teissier, 29680 Roscoff, France; European Research Institute for the Biology of Ageing, University Medical Center Groningen, University of Groningen, Groningen 9700AD, The Netherlands

**Keywords:** Acoela, Xenacoelomorpha, symbiogenesis, photosymbiosis, genome evolution

## Abstract

*Symsagittifera roscoffensis* is a well-known member of the order Acoela that lives in symbiosis with the algae *Tetraselmis convolutae* during its adult stage. Its natural habitat is the eastern coast of the Atlantic, where at specific locations thousands of individuals can be found, mostly, lying in large pools on the surface of sand at low tide. As a member of the Acoela it has been thought as a proxy for ancestral bilaterian animals; however, its phylogenetic position remains still debated. In order to understand the basic structural characteristics of the acoel genome, we sequenced and assembled the genome of aposymbiotic species *S. roscoffensis*. The size of *this* genome was measured to be in the range of 910–940 Mb. Sequencing of the genome was performed using PacBio Hi-Fi technology. Hi-C and RNA-seq data were also generated to scaffold and annotate it. The resulting assembly is 1.1 Gb large (covering 118% of the estimated genome size) and highly continuous, with N50 scaffold size of 1.04 Mb. The repetitive fraction of the genome is 61%, of which 85% (half of the genome) are LTR retrotransposons. Genome-guided transcriptome assembly identified 34,493 genes, of which 29,351 are protein coding (BUSCO score 97.6%), and 30.2% of genes are spliced leader trans-spliced. The completeness of this genome suggests that it can be used extensively to characterize gene families and conduct accurate phylogenomic reconstructions.

## Introduction

**Fig. 1.**
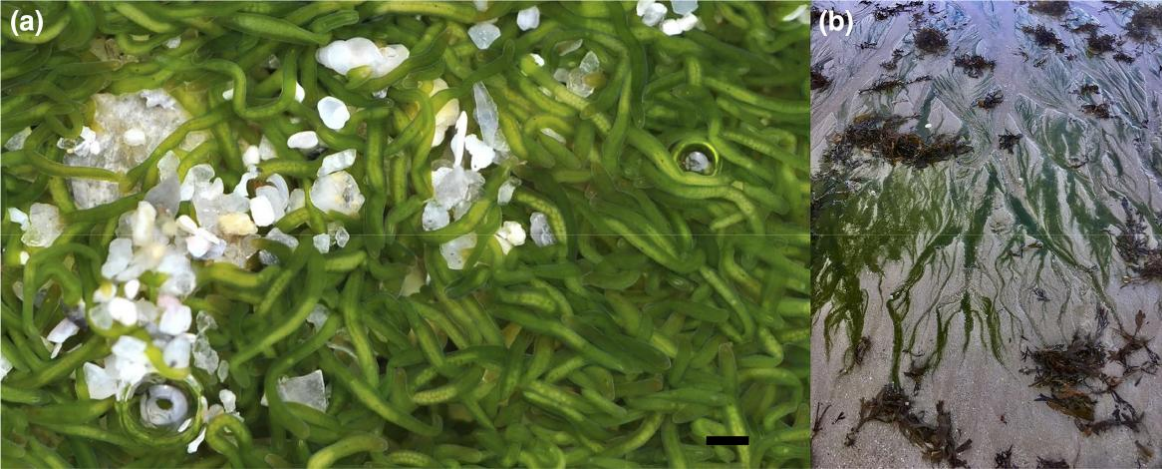
Specimens of the acoel *S. roscoffensis* in their natural habitat. a) Adult (gravid) *S. roscoffensis*. Credit Wilfried Thomas/Station Biologique de Roscoff. b) Biotope. Pools of adult specimens at low tide in a Brittany beach (France). Scale bar in panel a) is 1 mm.

Acoel flatworms (order Acoela) are members of the phylum Xenacoelomorpha, which also include the clades Nemertodermatida and Xenoturbellida (Philippe et al. 2011). The acoels are represented by approximately 400 described species, almost all of which are marine (Hejnol et al. 2009; Achatz et al. 2013). They exhibit a remarkable anatomical diversity, with many having salient characteristics such as an association with photosymbionts or extensive regenerative abilities. *Symsagittifera roscoffensis*, a species with the aforementioned properties, is 1 of the best-studied species of the Acoela (Fig. 1). Photosymbiotic adults are abundant along most of the Atlantic coast of Europe (from Wales to Gibraltar), easy to collect, and live in an obligatory relationship with the algae *Tetraselmis convolutae* (Bailly et al. 2014). As a member of the Acoela, a lineage considered to be an early offshoot of the Bilateria (but see Cannon et al. 2016; Philippe et al. 2019 for alternative views), it has also been used as models of ancestral bilaterians. Moreover, understanding the genomic characteristics of *S. roscoffensis* is of special relevance, as it can shed light on, for instance, symbiogenesis, regenerative processes, and, of course, the phylogenetic position of the Acoela.

In the recent past, we and others generated the first draft genome of *S. roscoffensis* (Philippe et al. 2019) but most contigs were extremely small (N50 < 5 kb). Here we present a new draft of the *S. roscoffensis* genome, generated using PacBio Hi-Fi technology and scaffolded with Hi-C data, which significantly increased genome assembly continuity (scaffold N50 = 1.04 Mb).

## Methods and materials

### Preparation of aposymbiotic animals

Aposymbiotic animals were used as a source of genomic DNA in order to avoid sequences of microalgae symbionts. Gravid animals were collected at low tide from beaches in the areas of Roscoff and Carantec, Brittany, France and were transported to laboratory, where most of them spontaneously spawned. Hatched juveniles were stored in RNAlater or snap-frozen in liquid nitrogen.

### Genome size measurement

The genome size was determined by a flow cytometry approach (Hare and Johnston 2011) as previously described for *Macrostomum lignano* flatworms (Wudarski et al. 2017). Nuclei were isolated from symbiotic adults and from juveniles without symbionts. The *M. lignano* NL12 line (Wudarski et al. 2017) and human fibroblasts were used as references. Nuclei were stained with propidium iodide and fluorescence was measured on a BD FacsCanto II Cell Analyzer.

### Pacific biosciences Hi-Fi genome sequencing

Genomic DNA was extracted using MagAttract High Molecular Weight DNA extraction kit. The preparation and sequencing of the library was performed by GenomeScan B.V. (Leiden, The Netherlands) using Sequel II Sequencing Kit 2.0 and 8M SMRT Cell. Reads were processed with the ccs tool v.6.2.0 and filtered using HiFiAdapterFilt v. 2.0.0 (Sim et al. 2022).

### Hi-C library construction and sequencing

The preparation and sequencing of Hi-C library was performed by Arima Genomics (San Diego, USA) using snap-frozen animals. The library was prepared using the Arima-HiC+ kit and the Arima Library Prep Module and sequenced on an Illumina HiSeq X instrument.

### RNA library construction and sequencing

RNA was isolated with Qiagen RNeasy Micro Kit according to the manufacturer's protocol, except that the DNase I treatment step was omitted. The RNA-seq library was constructed according to Smart-3SEQ protocol (Foley et al. 2019) and sequenced on an Illumina NextSeq 500 instrument.

### De novo transcriptome assembly

De novo transcriptome assembly SYMROS200831 was generated using a ReCAP pipeline (Grudniewska et al. 2016) from public whole transcript RNA-seq data (SRR5760179 and SRR8506641). Reads were normalized to 30× coverage and assembled into contigs using Trinity v.2.11.0 (Grabherr et al. 2011), remapped using Bowtie v.2.3.4.3 (Langmead and Salzberg 2012) and reassembled using CAP3 v. 12/21/07 (Huang and Madan 1999).

### Genome assembly and evaluation

PacBio Hi-Fi data were assembled with FALCON/FALCON-Unzip v.1.8.1/v.1.3.7 (Chin et al. 2016), Flye 2.9 (Kolmogorov et al. 2019), HiCanu v.2.2 (Nurk et al. 2020), Hifiasm v.0.16.1 (Cheng et al. 2021), IPA v.1.8.0 (Sovic 2022), Peregrine v.0.1.5.3 (Chin and Khalak 2019), Raven v.1.8.1 (Vaser and Šikić 2021), and wtdbg2 v.2.5 (Ruan and Li 2020); using parameters default for each assembler, and deduplicated by purge_dups v.1.2.5 (Guan et al. 2020). The quality of the assemblies was evaluated by mapping de novo transcriptome assembly SYMROS200831 to the genome assemblies with GMAP v.2021-08-25 (Wu and Watanabe 2005) and calculating the fraction of transcripts that map to a given assembly and the fraction of eukaryotic BUSCO models (v.2.0) present (Simão et al. 2015).

Hi-C reads were mapped to the deduplicated peregrine assembly by BWA v.0.7.17-r1188 (Li and Durbin 2009) and processed by the Arima Genomics mapping pipeline (https://github.com/ArimaGenomics/mapping_pipeline). Scaffolding was performed by SALSA2 v.2.3 (Ghurye et al. 2019). Paired-end RNA-seq reads (SRR5760179 and SRR8506641) were mapped to the scaffolds with HISAT v.2.2.1 (Kim et al. 2019) and further scaffolding was performed by P_RNA_scaffolder (Zhu et al. 2018). Gap closing was performed with LR_gapcloser v.1.1 (Xu et al. 2019) using initial PacBio Hi-Fi reads. Final polishing was done by pilon v.1.24 (Walker et al. 2014) with RNA-seq reads to fix frameshifts in coding sequences.

### Mitochondrial genome assembly and annotation

The mitochondrial genome was reconstructed by performing tblastn (Camacho et al. 2009) searches in genome assemblies generated by different assemblers using 11 protein-coding sequences from the *Isodiametra pulchra* mitochondrial genome (NC_034948.1). Two, 99.5% identical, candidates contigs were identified and further analyzed by self-blast. A single 100% identical terminal repeat (length 535) was identified and used for circularization. The resulting mitochondrial genome was annotated using MITOS2 web server (Donath et al. 2019).

### Repeat analysis

Tandem repeats were annotated with Tandem Repeat Finder 4.10.0 (Benson 1999). A de novo library of classified repetitive element models was created using RepeatModeler 2.0.3 (Flynn et al. 2020). Homology-based annotation of long terminal repeat (LTR) retrotransposons was done using the Domain-Associated Retrotransposon Search (DARTS) algorithm (Biryukov and Ustyantsev 2022). RepeatModeler- and LTR retrotransposon-derived libraries were merged and used as input for RepeatMasker 4.1.2-p1 (Tempel 2012).

### Gene prediction and annotation

Gene annotation was performed using TBONE pipeline (Wudarski et al. 2017). RNA-seq data SRR5760179 and SRR8506641 and Smart-3SEQ data generated in this study were mapped to the genome assembly with HISAT v.2.2.1 (Kim et al. 2019) and initial gene models were constructed by Scallop v0.10.5 (Shao and Kingsford 2017) and StringTie v2.2.1 (Kovaka et al. 2019). De novo transcriptome assembly SYMROS200831 was mapped to the genome assembly with GMAP v.2021-08-25 (Wu and Watanabe 2005). All gene models were merged by gffread v.0.12.7 (Pertea and Pertea 2020) and further processed to identify trans-spliced genes.

Trans-splicing leader sequence was determined through the analysis and mapping of the de novo transcriptome assembly SYMROS200831 to the genome assembly. Transcripts, in which first 10–50 nucleotides are not mapped to the genome, were identified and the respective nonmapped 5′ sequences were extracted from the transcripts. The most abundant sequences were manually aligned to each other and the trans-splicing leader sequence GCCTAATTGTTGTGATAAACTTATTAAATAGA was reconstructed. The structure of the spliced leader (SL) RNA gene was determined by mapping the SL sequence to the genome assembly using blastn (Camacho et al. 2009) and examining matching genomic regions for canonical SL RNA folding using RNAfold web server (Gruber et al. 2008).

Reads containing trans-splicing sequence were extracted from RNA-seq data, trimmed and mapped to the genome assembly with HISAT v.2.2.1 (Kim et al. 2019). The resulting wiggle files were used to identify genomic peaks corresponding to trans-splicing locations. Similarly, peaks corresponding to polyadenylation sites at 3′ end gene boundaries were identified by mapping reads from Smart-3SEQ RNA-seq libraries. The generated trans-splicing and polyadenylation signals were used to refine gene boundaries and separate trans-spliced genes. Open reading frames were predicted by TransDecoder v.5.5.0 (Haas et al. 2013). To remove redundancy, for each genomic locus a single representative transcript was selected and included into a subset called “core genes.”

## Results and discussion

### Genome assembly and evaluation

In order to distinguish the genomes of *S. roscoffensis* and its microsymbiotic algae, we used cultured aposymbiotic juveniles without microsymbionts. Using a flow cytometry approach, the genome size of *S. roscoffensis* is estimated to be in the range of 910–940 Mb (Supplementary Fig. 1). We sequenced the genome to 20× coverage with Pacific Biosciences Hi Fidelity reads (1.29 mln ccs reads, mean length 15.7 kb) and assembled the data with 8 different genome assemblers (Supplementary Table 1). For the evaluation of the assemblies, we examined: the assembly size, N50 contig length, the fraction of transcripts from de novo transcriptome assembly mapping to the genome, and the number of gene models from the Eukaryotic BUSCO subset identified. We focused specifically on the Eukaryotic BUSCOs because almost all genes from this subset are expected to be present in an Acoel genome. Since genome sequencing was performed on a population of animals obtained directly from a natural habitat, it is expected that a substantial level of heterozygosity is present in the sequencing data, leading to large assemblies with under-collapsed heterozygous regions. Indeed, FALCON, Flye, Hifiasm, IPA, and Peregrine produced redundant assemblies of 1.2–2.5 Gb in size (Supplementary Table 1). HiCanu and Raven over-collapsed the assemblies (737 and 555 Mb), while Wtdbg2 generated an assembly closest in size to the measured genome size (935 Mb). Wtdbg2 also produced the highest N50 length (140.3 kb) among the tested assemblers. Moreover, the fraction of de novo transcripts mapped and BUSCO models identified was the highest for the Peregrine assembly (Supplementary Table 1). After deduplication of the redundant assemblies with purge_dups (Guan et al. 2020), the Peregrine assembly appeared to be substantially better compared to all other tested assemblies, producing a N50 size of 197.6 kb and the lowest fraction of missing de novo and BUSCO transcripts, while its assembly size of 1.1 Gb is only ∼18% larger than the measured genome size (Supplementary Table 1). Therefore, Peregrine assembly was used for further scaffolding and gap closing. We deliberately choose an under-collapsed rather than an over-collapsed assembly in order to maximally retain gene content, although this means that the assembly does contain some regions that represent diverged alleles and not true genomic duplications.

For genome scaffolding, we generated 388 mln Illumina read pairs (∼100× genome coverage) from a Hi-C library. Scaffolding was performed by SALSA2 (Ghurye et al. 2019), followed by P_RNA_scaffolder (Zhu et al. 2018) with RNA-seq reads, which substantially improved assembly continuity (3,460 scaffolds, N50 = 1039.9 kb, Table 1). Gaps were closed by LR_gapcloser (Xu et al. 2019) followed by assembly polishing with pilon (Walker et al. 2014), reducing the number of contigs from 8,943 to 7,843 and improving N50 contig size from 197.6 to 237.9 kb (Table 1). The mitochondrial genome was reconstructed from PacBio Hi-Fi genome assemblies and is 99% identical to the published sequence (Mwinyi et al. 2010).

**Table 1.** Characteristics of genome assembly SymRos_1_5.

	Contigs	Scaffolds
Total number	7,843	3,460
Total length (bp)	1,101,399,379	1,103,025,803
Average length (bp)	140,431	318,794
Shortest (bp)	12,747	12,747
Longest (bp)	1,836,468	8,003,794
N50 (bp)	237,875	1,039,899
L50	1,417	287
GC content (%)	36.7	36.7
		
Coding genes		29,351
Noncoding genes		5,142
Number of SL trans-spliced genes		10,433
Average transcript length (kb)		1.78
Longest transcript (kb)		53.5
Average gene length (kb)		9.83
Average number of introns		3.2
Average intron length (kb)		2.5
		
Eukaryotic BUSCOs (*n* = 303)		296 (97.6%)
ȃComplete and single-copy BUSCOs		188 (62%)
ȃComplete and duplicated BUSCOs		108 (35.6%)
Fragmented BUSCOs		3 (1.0%)
Missing BUSCOs		4 (1.4%)

### Repeat annotation

The genome is highly repetitive, with transposable elements and simple repeats comprising more than 60% of its sequence, of which 85% are LTR retrotransposons (Supplementary Table 2).

### Gene annotation

To annotate genes, we used TBONE pipeline (Wudarski et al. 2017), which takes into account potential effects of SL trans-splicing present in flatworms (Ustyantsev and Berezikov 2021). By analyzing de novo transcriptome assembly SYMROS20083, we determined that SL trans-splicing is also present in *S. roscoffensis* (Supplementary Fig. 2). The genome-guided transcriptome assembly SymRos_1_5_RNA.v1 generated by TBONE pipeline contains 34,493 genes, of which 29,351 are protein-coding and 5,142 are noncoding (Table 1). The number of SL trans-spliced genes is 10,433, comprising 30.2% of all genes. The transcriptome assembly contains 296 out of 303 eukaryotic BUSCO gene models, or 97.6% (Table 1).

Preliminary analysis confirmed the presence of gene families (Homeobox classes, bHLHs, GPCRs and Wnts; with their rich complements) described in previous papers (Moreno et al. 2009; Gavilán et al. 2016; Brauchle et al. 2018). This, again, attests the quality and usefulness of the genome assembly. At the same time, the number of duplicated BUSCO genes is quite high at 35.6% (Table 1). Some of these duplications might be true, since partial genome duplications have been reported in flatworms (Zadesenets et al. 2017), but these duplications can be also explained by the fact that we used an under-collapsed assembly for scaffolding. False gene duplications are a known and difficult to address issue in genome assemblies, stemming from the inability of genome assembly algorithms to discriminate between haplotype paralogs and homologs in highly heterozygous regions (Ko et al. 2022). Thus, the *S. roscoffensis* genome assembly reported here should be used with caution when analyzing potential gene family expansions.

### Characteristics of the *S. roscoffensis* genome and comparison with other xenacoelomorphs

Here, by combining PacBio Hi-Fi sequencing with Hi-C scaffolding, we generated a highly continuous and complete assembly SymRos_1_5 with N50 scaffold size of 1.04 Mb and BUSCO score of 97.4. The karyotype of *S. roscoffensis* is 2*n* = 20 (Moreno et al. 2009). Despite the availability of Hi-C data, the assembly is still far from chromosome-level, which can be attributed to the highly repetitive nature of the genome and high level of heterozygosity in the population of animals used.

Based on flow cytometry data the genome size of *S. roscoffensis* is in the range of 910–940 Mb, which is comparable to that of other acoel genomes, *Hofstenia miamia* (950 Mb) (Gehrke et al. 2019) and *Praesagittifera naikaiensis* (654 Mb) (Arimoto et al. 2019). The assembled genome is larger than the measured genome size, likely due to remaining heterozygous regions not purged from the assembly.

The GC content of *S. roscoffensis* genome is 36.7% (Table 1), thus it is an AT-rich genome, similar to other xenacolomorphs [43% GC content in *Xenoturbella bocki* (Schiffer et al. 2022), 39.1% GC content in *P. naikaiensis* (Arimoto et al. 2019)].

In the assembled genomes of acoels, the content of repetitive sequences is high and varies from 53% in *H. miamia* (Gehrke et al. 2019) to 61% in *S. roscoffensis* and 70% in *P. naikaiensis* (Arimoto et al. 2019), with the major prevalence of LTR retrotransposons in all of them. In contrast, the xenoturbellid *X. bocki* has only 25% of its genome in repeats (Schiffer et al. 2022).

We annotated 34,493 genes in *S. roscoffensis*, which is higher than those reported for *H. miamia* (∼22,000) or *X. bocki* (∼15,000). This variability can be explained by differences in annotation pipelines used to identify genes, with the TBONE pipeline used here more inclusive for nonconserved, noncoding, repetitive, and low-expressed genes.

Some organisms, including flatworms, have SL trans-splicing, in which sequence from 1 RNA molecule (SL) is spliced to 5′ ends of different mRNAs (Lasda and Blumenthal 2011). We identified that 30.2% of the genes in *S. roscoffensis* undergo such SL trans-splicing (Table 1), which is similar to the number of trans-spliced genes in the flatworm *M. lignano* (Ustyantsev and Berezikov 2021).

## Supplementary Material

jkac336_Supplementary_Data

## Data Availability

All raw sequencing data have been deposited in the NCBI Sequence Read Archive (accession codes SRR20990873–SRR20990875) and can be accessed with BioProject No. PRJNA867535. The genome assembly has been deposited at DDBJ/ENA/GenBank under the accession JANVAR000000000. The annotated genome is available at http://gb.macgenome.org. Supplemental material available at G3 online.

## Notes

*Communicating editor: M. Nowrousian*
