# Supplementary material for: Genome assembly of the acoel flatworm Symsagittifera roscoffensis, a model for research on body plan evolution and photosymbiosis
Source: G3 (Bethesda). 2022 Dec 21;13(2):jkac336. doi: 10.1093/g3journal/jkac336 (PMC9911081; doi:10.1093/g3journal/jkac336)
Supplement: jkac336_Supplementary_Data [file jkac336_supplementary_data.pdf]

Supplementary Data

Genome assembly of the acoel flatworm *Symsagittifera roscoffensis,* a model for research on body plan evolution and photosymbiosis

Pedro Martinez, Kirill Ustyantsev, Mikhail Biryukov, Stijn Mouton, Liza Glasenburg, Simon G. Sprecher, Xavier Bailly and Eugene Berezikov

Supplementary Figure 1. Genome size measurement of *S. roscoffensis.*

Supplementary Figure 2. SL trans-splicing in *S. roscoffensis.*

Supplementary Table 1. Statistics of the initial genome assemblies.

Supplementary Table 2. Repeat content of SymRos_1_5 genome assembly

A

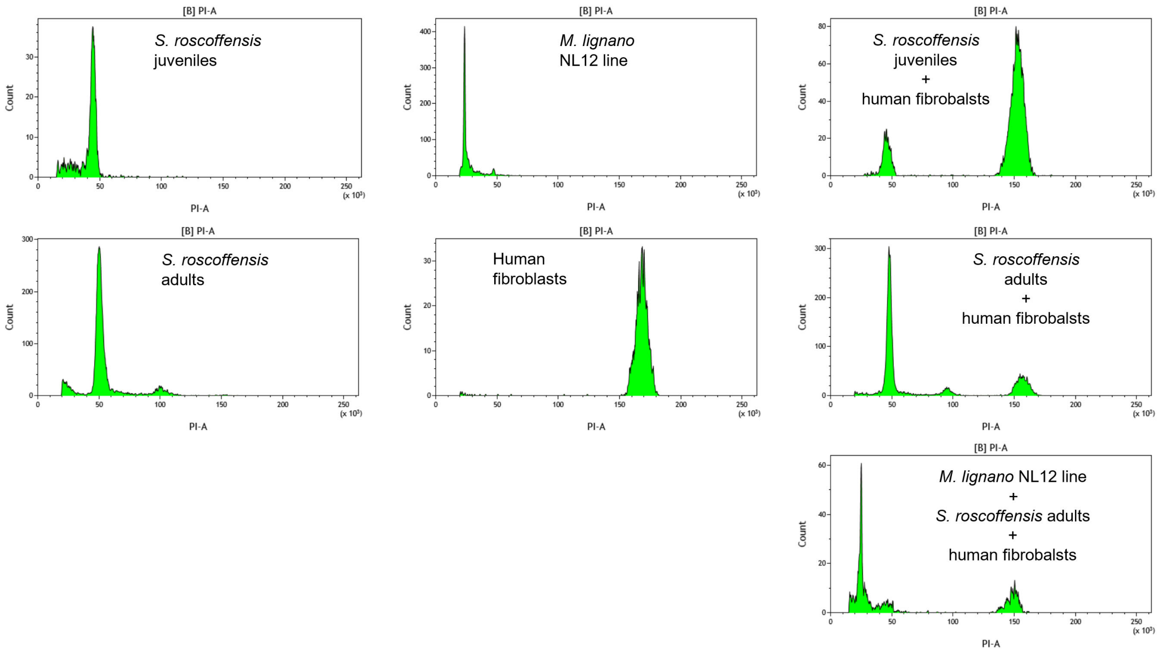

B

	Intensity			Calculated genome size			
	**Human**	***S. roscoffensis***	***M. lignano***		**Human**	***S. roscoffensis***	***M. lignano***
NL12+Juv+Human	149.2	46.2	25.0	Total pg	6.1987	1,92	1.04
				Total Mb	6062.35	1877.22	1015.81
				Haploid Mb	3031.18	**938.61**	507.90
Juv + Human	152.7	45.9		Total pg	6.1987	1.86	
				Total Mb	6062,35	1822.28	
				Haploid Mb	3031.18	**911.14**	
Adults +Human	156.7	48.1		Total pg	6.1987	1.90	
				Total Mb	6062.35	1860.87	
				Haploid Mb	3031.18	**930.44**	

Supplementary Figure 1: Genome size measurement of *S. roscoffensis.* (*A*) Separate and combined measurements of fluorescence in *S. roscoffensis*, *M. lignano* NL12 line and human fibroblasts. (*B*) Calculation of *S. roscoffensis* genome sizes using human fibroblasts as a reference and *M. lignano* NL12 line as a positive control.

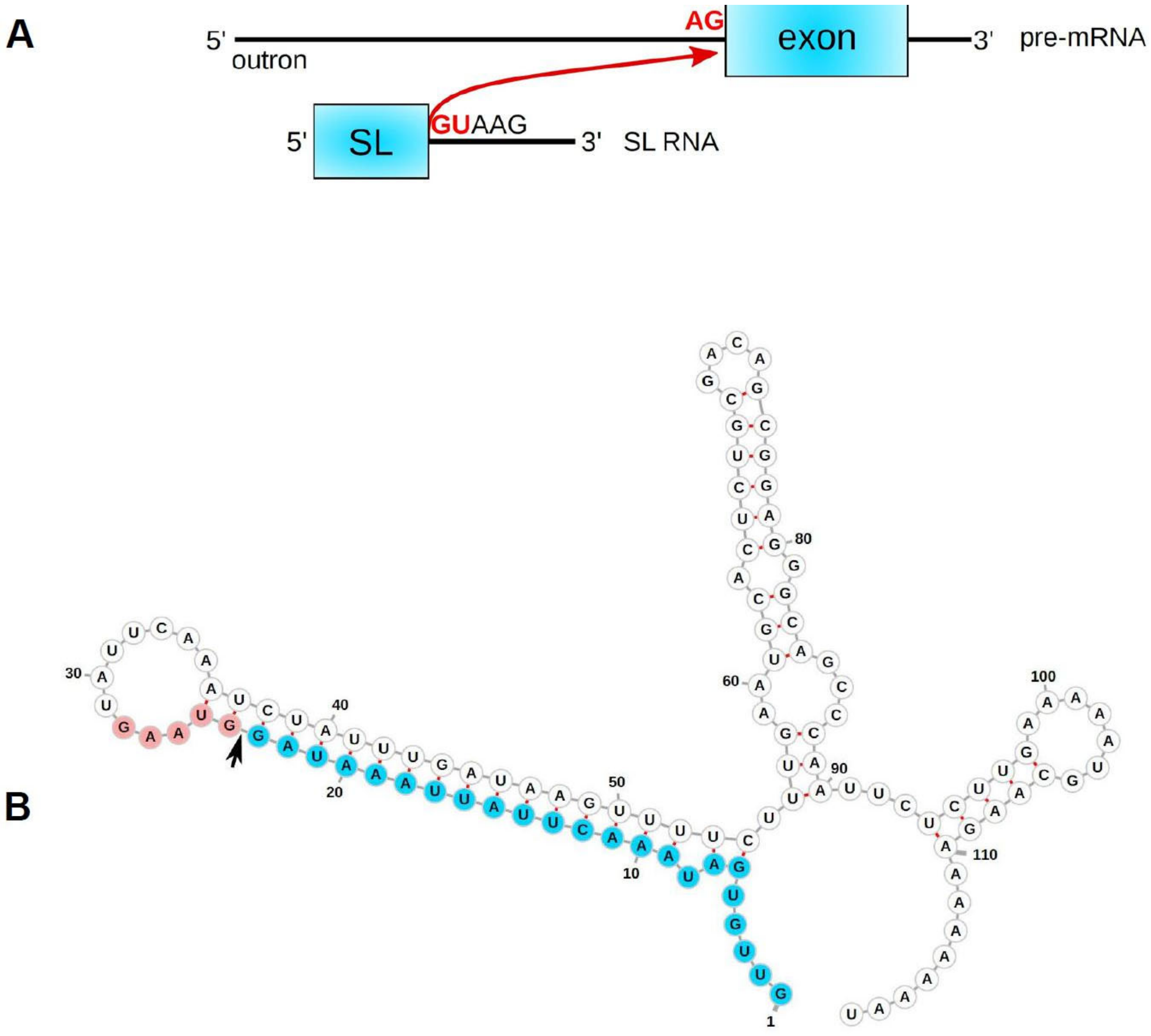

Supplementary Figure 2. SL trans-splicing in *S. roscoffensis.* (*A*) General principle of SL trans-splicing. Spliced leader (SL) sequence from SL RNA is trans-spliced to an exon of a target pre-mRNA. (*B*) Predicted secondary structure of putative *S. roscoffensis* SL RNA. Spliced leader sequence is show in blue. Arrow indicates the site of trans-splicing.

Supplementary Table 1. Statistics of the initial genome assemblies.

**Assembler**	**Full assembly**						**Deduplicated assembly**					
	**Size (Mb)**	**N50 (Kb)**	***De novo* transcripts (%)**		**BUSCO models (%)**		**Size (Mb)**	**N50 (Kb)**	***De novo* transcripts (%)**		**BUSCO models (%)**	
			**F**	**M**	**F**	**M**			**F**	**M**	**F**	**M**
FALCON	1,582	86.1	8.1	12.4	4.7	4.0	961	135.6	9.6	16.8	5.0	10.0
Flye	1,243	52.9	9.2	14.5	5.3	3.7	832	63.0	11.0	19.6	6.7	9.3
HiCanu	737	50.2	13.8	32.4	15.0	22.7	*737*	*50.2*	*13.8*	*32.4*	*15.0*	*22.7*
Hifiasm	1,744	68.6	12.5	23.7	13.7	13.7	798	83.3	12.7	25.1	13.3	14.7
IPA	2,005	71.8	5.6	9.0	3.0	1.3	1,172	79.4	9.0	12.0	9.3	6.0
Peregrine	2,537	83.9	**4.2**	**7.9**	**0.7**	**1.3**	1,101	**197.6**	**6.5**	**9.3**	**1.3**	**1.3**
Raven	555	100.0	12.1	14.4	10.7	5.0	*555*	*100.0*	*12.1*	*14.4*	*10.7*	*5.0*
Wtdbg2	**935**	**140.3**	8.0	10.6	2.7	4.3	***935***	*140.3*	*8.0*	*10.6*	*2.7*	*4.3*

F = Fragmented, M = Missing

Supplementary Table 2. Repeat content of SymRos_1_5 genome assembly

**Repeat group**	**% of the genome**
DNA transposons	3.88
Non-LTR retrotransposons (LINEs)	1.68
LTR retrotransposons	51.75
SINEs	0.02
Low complexity and simple repeats	1.18
Tandem repeats by TRF	2.63
**Total**	**61.14**
